# Impact of dietary fiber on gut microbiota composition, function and gut-brain-modules in healthy adults – a systematic review protocol

**DOI:** 10.12688/hrbopenres.13794.1

**Published:** 2023-10-20

**Authors:** David Antoine Lachmansingh, Benjamin Valderrama, Thomaz Bastiaanssen, John Cryan, Gerard Clarke, Aonghus Lavelle

**Affiliations:** 1 Department of Psychiatry and Neurobehavioural Science, University College Cork, Cork, County Cork, Ireland; 2 APC Microbiome Ireland, University College Cork, Cork, County Cork, Ireland; 3 Department of Anatomy and Neuroscience, University College Cork, Cork, County Cork, Ireland

**Keywords:** Microbiome, dietary fiber, gut microbes, healthy individuals, fiber intervention

## Abstract

**Background:** The gut microbiota has been implicated in health and disease. Its functional outputs such as microbial metabolites, are considered important in this regard. This includes the production of neurochemicals/neuroactives which are associated with microbial regulation of central nervous system (CNS) function, potentially facilitating mood regulation. Significant associations exist between alterations in the relative abundance of specific microbial taxa and psychiatric disorders. Communication between the gut microbiota, the gastrointestinal (GI) tract and the CNS occurs via the microbiota-gut-brain axis bidirectionally. Here, dietary fiber may have an impact, potentially altering gut microbiota composition and function, modifying bacterial enzymatic function and metabolite production. As many taxa of microorganisms have enzymes capable of producing or degrading neurochemicals i.e. neuroactive gut brain modules (GBMs), new predictive tools can be applied to existing datasets such as those harvested from dietary fiber interventions. We will perform a systematic review in order to identify studies reporting compositional gut microbiota alterations after interventions with dietary fiber in healthy individuals. We aim to extract publicly available microbial genomic sequence datasets for reanalysis, with the intention of identifying altered GBMs following dietary fiber interventions.

**Methods:** Interventional trials and randomized controlled studies that are originally published involving healthy adult humans will be included. A systematic search of PubMed/MEDLINE and EMBASE, two electronic databases, will be completed.

**Discussion:** Dietary fiber impacts gut microbiota composition, promoting the growth of particular taxa, while others are reduced in relative abundance. Our search focuses on the impact of this food component on the microbiota of healthy individuals. Our review will compile and update compositional gut microbial changes after reanalysis of their datasets with a consistent bioinformatic pipeline. More detailed functional consequences in terms of neuroactive GBMs may be predicted.

**Registration:** Prospective registration with PROSPERO (International Prospective Register of Systematic Reviews) – registration no. CRD42023437655.

## Introduction

The gut microbiota is the immense collection of bacteria, viruses, eukaryotes and archea within our gastrointestinal (GI) tract ( Butler *et al.*, 2019; Lurie-Weinberger & Gophna, 2015; Matijašić *et al.*, 2020). It has been described as a “forgotten organ”, with numerous critical functions as well as implications in health and disease ( Clarke *et al.*, 2014; Sun *et al.*, 2022). Breaking down complex carbohydrates whilst producing short chain fatty acids ( O’Riordan *et al.*, 2022) and providing defence against pathogenic species and infections ( Wiertsema *et al.*, 2021), are just a few of the essential benefits of these organisms. Additionally, the gut microbiota aids in the production of a number of neurochemicals and other neuroactives, and modifies the availability of their precursors, resulting in important gut-brain axis signalling and CNS function ( Valles-Colomer *et al.*, 2019). There is evidence to suggest that it may play a role in mood regulation both in animal and human clinical studies ( Bastiaanssen *et al.*, 2019; Kelly *et al.*, 2016). More recently, it was found that there were strong associations between the gut microbiota and depressive symptoms ( Bosch *et al.*, 2022) and more specificially, several microbial taxa were associated with depressive symptoms in individuals ( Radjabzadeh *et al.*, 2022). Alterations in microbial taxa within the gut have also been associated with increased externalizing behavior at the age of 10, a risk factor for the developing mental health disorders and risky lifestyles later in adulthood ( Ou *et al.*, 2022).

The gut microbiota, along with the GI tract and CNS, as well as communication conduits such as the autonomic nervous system, the enteric nervous system, the hypothalamic-pituitary-adrenal (HPA) axis and the immune system comprise the microbiota-gut-brain axis ( Cryan *et al.*, 2019). Being very complex, communication along the microbiota-gut-brain axis is bidirectional ( Järbrink-Sehgal & Andreasson 2020). Gut micro-organisms and their metabolites may stimulate vagal afferent fibres, resulting in direct transmission of signals to the CNS ( Berding *et al.*, 2021). Other microbial products may reach the brain in an “endocrine” fashion via the blood circulation and crossing the blood brain barrier. Likwise the opposite is possible, whereby the brain may influence the GI tract and it’s associated microbiota via the circulation and also by way of neuronal pathways of the vagus nerve. Disturbances in brain-gut-microbiota communication may influence eating behaviors, resulting in consumption of foods which have the potential to alter the gut microbiome ( Gupta *et al.*, 2020).

Dietary fiber, defined as edible, non-digestible (resistant to endogenous digestive enzymes), mostly plant-derived carbohydrate polymers consisting of at least 3 monomeric units ( ISAPP, 2018; Makki *et al.*, 2018), has numerous benefits to health. These include cardiovascular, gastrointestinal and mental health benefits just to mention a few ( Barber *et al.*, 2020). It is thought that many of these beneficial functions are exhibited via the gut microbiome ( Lancaster *et al.*, 2022). Dietary fiber has the potential to alter gut microbiota composition and function and promotes the production of metabolites such as SCFAs ( Barber *et al.*, 2020; Myhrstad *et al.*, 2020). High fiber diets appear to facilitate microbiota enzymatic function leading to an immune response that is dependent on the individual ( Wastyk *et al.*, 2021).

Apart from SCFAs, bacterial enzymes produce numerous other biomolecules, many of which are relevant to brain function and behavior. This is encompassed within the concept of gut brain modules (GBMs). GBMs are specific pathways, within each of which there is either production or degradation of a particular neurochemical requiring the action of specific enzymes ( Spichak *et al.*, 2021; Valles-Colomer *et al.*, 2019). These pathways are thought to be integral to microbiota-gut-brain axis communication and involve specific microbes. In order to fulfill the criteria for a GBM, the micro-organism must possess each enzyme within the specified pathway ( Spichak *et al.*, 2021; Valles-Colomer *et al.*, 2019). The gut microbiota, in this regard may therefore be involved in the regulation of certain neurotransmistters or their precursors and other molecules that can potentially have an effect on the CNS. This GBM framework can be incorporated into new predictive tools applied to existing datasets.

Sequencing technologies for DNA/RNA have aided in the identification of particular taxa or species within fecal samples and allow for not only compositional analysis, but also functional analysis of the microbiome ( Bastiaanssen *et al.*, 2019). Two popular techniques are 16S rRNA gene/transcript sequencing and whole genome “shotgun” sequencing (WGS) ( Bastiaanssen *et al.*, 2019; Ranjan *et al.*, 2016). Many published studies investigating compositional and functional changes in the gut microbiota e.g., after an intervention, have yielded publicly available microbiome datasets thanks to high-throughput sequencing technologies ( Liu *et al.*, 2021). Once retrieved, these datasets can then be processed through software programs and their sequences mapped to reference databases, with their taxonomy changes and/or function inferred ( Bastiaanssen *et al.*, 2022).

We intend to perform a systematic review to identify compositional gut microbiota alterations after interventions with forms of dietary fiber in healthy individuals. We aim to not only provide a narrative comparison of the compositional changes between studies, but also to select studies with publicly available datasets to reanalyse and identify predicted fiber-induced alterations in GBMs. This will allow us to provide a functional account of the gut microbial compositional alterations following dietary fiber interventions.

### Objectives

Our primary research question is: In healthy individuals, is dietary fiber associated with a change in microbiota composition and/or function in comparison to a control group or participant pre-intervention? A secondary research question is to determine if a dietary fibre intervention has predicted beneficial effects on GBMs.

To address these questions, we have outlined the following objectives:

1. To systematically review and reanalyse published studies that have investigated the effects of a dietary fiber intervention on the gut microbiome in healthy individuals.

2. To undertake a reanalysis of microbiome sequencing data from these studies with a consistent bioinformatic pipeline to determine if GBMs are altered by a dietary fiber intervention.

## Methods

The systematic review will be conducted in accordance with the guidelines for the Preferred Reporting Items for Systematic Review and Meta-analysis Protocols (PRISMA-P) ( Moher *et al.*, 2015). Following the PICO framework, the population includes healthy individuals over 18 years of age, whereas the intervention would be dietary fiber. Depending on the study, the control would either be the within-study control group or participant pre-intervention. The outcome would be compositional and functional microbiota alterations.

### Eligibility criteria

Original studies that are interventional trials and randomized controlled studies (both cross-over and non-crossover) will be included in this systematic review. Studies will include interventions in the form of specific dietary fiber supplementation and also whole diets where fiber intake is increased. Additionally, studies will include healthy, human adults, and will also have reported microbiome analyses. Only those with microbiome data obtained from 16S or WGS methods of sequencing will be included. However, the absence of available sequence datasets within studies will not be part of the exclusion criteria for this search. Nevertheless, such studies without sequence datasets will not be applied to our downstream analyses. Publications that are reviews, case reports and expert opinions will not meet the eligibility criteria and therefore will not be included. However, their bibliographies will nonetheless be searched in turn for eligible studies. 

### Information sources and search strategy

Two electronic databases, i.e., PubMed/MEDLINE and EMBASE will be systematically searched. Only peer-reviewed publications from 2010–2023 will be considered. In terms of search vocabulary, medical subject heading (MeSH) terms will be used. We propose our search strategy to be as follows:

(fibre OR fiber OR ‘dietary fiber’ OR ‘dietary fibre’ OR ‘whole grain’) AND (‘microbiome’ OR ‘microbiota’ OR ‘microflora’ OR ‘metagenomic*’ OR 16S) AND (intervention OR trial OR ‘randomized control* trial’ OR ‘randomised control* trial’)

### Selection of studies

Two reviewers (DAL and AL) will screen titles and abstracts of studies independently after conducting the initial literature search as described above. Studies that do not meet the criteria for eligibility will be screened and excluded. For example, studies that examined the effect of dietary fiber on the microbiota in pathological states would be identified in the initial search attempt but then excluded in order to refine the search results to include healthy study populations. Potentially eligible studies, including their full text, will be screened by the same reviewers. Should there be a discrepancy at this stage, a 3 ^rd^ reviewer (GC) will mediate. Construction of a PRISMA flow diagram ( Page *et al.*, 2021) will be done in an attempt to illustrate the articles examined at each stage. Details such as the number of papers included and excluded, as well as the reasons for exclusion of the latter will be noted on this diagram.

### Data extraction and management

The reviewers DAL and AL will extract data from the selected studies. This will include reference citations, population description, types of dietary fiber intervention, study design, study size (both initial numbers and intention to treat), year of publication as well as other study and result characteristics, including microbiome alterations and clinical changes in response to the intervention. Some important data to be further extracted from selected studies required for our secondary objective, will include the type of sequencing technology used (e.g., 16S or WGS) and links to datasets within online repositories e.g., the National Center for Biotechnology Information (NCBI) or the European Bioinformatics Institute (EBI).

Studies will be tabulated in a format depicting the abovementioned characteristics.

### Outcome data

The primary outcome will be changes in microbiome parameters, such as alpha and beta diversity, taxonomic composition and microbiome function in response to a dietary fibre intervention. As part of our secondary objective, we will obtain differential abundances between treatment groups, as well as data relating to alterations in GBMs with different fiber interventions.

### Data synthesis

We will endeavour to group studies with similar compositional outcomes and other key outputs. Additionally, as described above, the summary characteristics of all included articles will be displayed in tables. The tables will include key features of the studies such as details of study populations (initial N and intention to treat), study design and type/form of dietary fiber intervention. Outcomes in the form of alterations of abundance of specific taxa/species and/or diversity within each study included in this secondary research review, will also be represented within the mentioned tables.

If genomic sequence datasets are available, we will reanalyse these separately using a consistent bioinformatic pipeline which we hope may not only help update the compositional alterations, but also assist in predicting changes in GBM function with respective dietary fiber interventions.

## Ethics

As this study entails secondary research, and will not involve any animals or human subjects, ethical approval is not required. The gathering, accessing, processing or use of personal information that could potentially identify individuals will not occur. Some of the results from this systematic review will be used in further statistical analysis as explained previously.

## Discussion

Different studies have suggested that various types of dietary fiber have an impact on the gut microbiota in terms of promoting compositional changes ( Guan *et al.*, 2021; Holscher, 2017). Some types of dietary fiber may promote certain taxa ( Holscher *et al.*, 2015). Other types of fiber may promote some taxa whilst reducing others ( Kaczmarek *et al.*, 2019; Ranaivo *et al.*, 2022). An assortment of studies has examined the effect of dietary fiber on the gut microbiota in pathological states e.g. obesity ( Mocanu *et al.*, 2021) and ulcerative colitis ( Fritsch *et al.*, 2021). However, our interest lies in examining studies reporting the effects of dietary fiber interventions on the gut microbiota of healthy individuals.

By refining our search to only involve studies with healthy populations, we aim to achieve a greater appreciation for the baseline changes seen with dietary fiber interventions. Such restriction will help control or account for confounding factors that are evident in disease states ( Jager *et al.*, 2008). This approach will help us to not only update and confirm the compositional alterations, but also uncover the possibility of other genera of micro-organisms that may not have been accounted for during initial analysis. This is plausible, given the differences with software technologies, techniques and human expertise ( Nearing *et al.*, 2022; Tsou *et al.*, 2020) as well as database updates since the studies were published.

The concept of reanalyzing datasets is not new. It has been used to uncover aspects of dysregulated immune responses to COVID-19 infection, as part of the infectious disease mechanism ( Alberts *et al.*, 2022). The technique has proved useful in terms of understanding the host response as well as identifying further candidates with diagnostic potential in other infections such as leprosy ( Leal-Calvo & Moraes, 2020). It is thought that reanalysis of datasets is a potentially useful tool in the research of rare diseases and allows for the discovery of genetic disease variants ( Setty *et al.*, 2022). Importantly, reanalysis of datasets has been applied to microbiota-gut-brain axis research in which evidence of disease-related microbial metabolic pathway alterations were uncovered in illnesses such as Alzheimer’s Disease, schizophrenia, anxiety and depression ( Spichak *et al.*, 2021).

However, our intended use of dataset reanalysis, especially in terms of predicting GBM function in relation to dietary fiber interventions, to our knowledge has not been done before. There will be limitations to our study as we expect significant heterogeneity between selected studies. We anticipate that there will be significant variance not only in study methodology, but also in terms of dietary intervention types, healthy population characteristics (e.g. geographical location, ethnicity, sex), study design and methodology as well as compositional outcomes.

## Study status

At this moment, our preliminary searches have been completed and our selection of studies and formal screening are in progress. Data extraction, synthesis and tabulation are to follow.

## Data Availability

No data available as this is a protocol. Zenodo. PRISMA-P Reporting Checklist and PRISMA Flow Diagram for “Impact of dietary fiber on gut microbiota composition, function and gut-brain-modules in healthy adults – a systematic review protocol”. DOI: https://doi.org/10.5281/zenodo.8306848 Data are available under the terms of the Creative Commons Attribution 4.0 International Public License https://creativecommons.org/publicdomain/zero/1.0/ (Attribution 4.0 International). GC, DAL and JFC initiated the research whereas DAL, BV, TB and AL contributed to the design of the protocol. GC contributed towards the protocol’s revision. DAL completed the pilot search, while AL, GC and DAL will be involved in the formal selection of studies. DAL and AL will perform the data extraction. DAL, BV, TB, AL, GC and JFC will draft the manuscript. All of the authors contributed to the review as well as revision of the manuscript and approved the publication.
