# Supplementary material for: Impact of dietary fiber on gut microbiota composition, function and gut-brain-modules in healthy adults – a systematic review protocol
Source: HRB Open Res. 2024 Feb 26;6:62. Originally published 2023 Oct 20. [Version 2] doi: 10.12688/hrbopenres.13794.2 (PMC10958149; doi:10.12688/hrbopenres.13794.2)
Supplement: Peer review history

# Reviewer report: Reviewer response for version 2

**DOI:** 10.21956/hrbopenres.15197.r38333

**Published:** 2024-03-21

**Authors:** Evelyn Medawar

**Affiliations:** 1 Max Planck Institute for Human Cognitive and Brain Sciences, Leipzig, Germany

The authors have revised the protocol in an acceptable way. Well done.

Is the study design appropriate for the research question?

Yes

Is the rationale for, and objectives of, the study clearly described?

Yes

Are sufficient details of the methods provided to allow replication by others?

Partly

Are the datasets clearly presented in a useable and accessible format?

Not applicable

Reviewer Expertise:

gut-brain communication, plant-based diets, dietary decision-making

I confirm that I have read this submission and believe that I have an appropriate level of expertise to confirm that it is of an acceptable scientific standard.

# Reviewer report: Reviewer response for version 1

**DOI:** 10.21956/hrbopenres.15092.r37631

**Published:** 2024-01-16

**Authors:** Evelyn Medawar

**Affiliations:** 1 Max Planck Institute for Human Cognitive and Brain Sciences, Leipzig, Germany

The protocol is very neatly structured and well descibres the research question and method.

 The topic is highly relevant for moving research in the gut-brain communication field further.

 The methods are adequately chosen and described.

 There are some questions I would like to see replied to:

 - "healthy individuals": please define healthy and aim to report the study population descriptives as per study (e.g. will an overweight sample be included yes or no?)

 - fibre intervention or dietary fiber diet should be reported as per study (as study descriptive) and in detail described as well as length of intervention

 - gut-brain-module: needs further precision for what is meant and how these are defined and selected and by what method

Is the study design appropriate for the research question?

Yes

Is the rationale for, and objectives of, the study clearly described?

Yes

Are sufficient details of the methods provided to allow replication by others?

Partly

Are the datasets clearly presented in a useable and accessible format?

Not applicable

Reviewer Expertise:

gut-brain communication, plant-based diets, dietary decision-making

I confirm that I have read this submission and believe that I have an appropriate level of expertise to confirm that it is of an acceptable scientific standard, however I have significant reservations, as outlined above.

# Reviewer report: Reviewer response for version 1

**DOI:** 10.21956/hrbopenres.15092.r36914

**Published:** 2023-11-27

**Authors:** Chris Gill

**Affiliations:** 1 Nutrition Innovation Centre for Food and Health (NICHE), Ulster University, Coleraine, Northern Ireland, UK

A comprehensive summary of study protocol is provided detailing approach to understanding the impact of dietary fiber on gut microbiota composition, function and gut-brain-module. The approach is well structured and consistent with the question to be asked.

 The introduction mentions a definition of fibre is this to be applied in the SR. A minor point for consideration, consider inclusion of definition of how the term fibre is applied in any given study, fibre quantified based on what method or source, which methods was applied to quantify.

Is the study design appropriate for the research question?

Yes

Is the rationale for, and objectives of, the study clearly described?

Yes

Are sufficient details of the methods provided to allow replication by others?

Yes

Are the datasets clearly presented in a useable and accessible format?

Yes

Reviewer Expertise:

Gut health, digestion, polyphenols, bioactivity, human intervention trials,

I confirm that I have read this submission and believe that I have an appropriate level of expertise to confirm that it is of an acceptable scientific standard.
